# Linear regression model for metal–organic frameworks with CO_2_ adsorption based on topological data analysis

**DOI:** 10.1038/s41598-024-62858-7

**Published:** 2024-05-26

**Authors:** Kazuto Akagi, Hisashi Naito, Takafumi Saikawa, Motoko Kotani, Hirofumi Yoshikawa

**Affiliations:** 1 Advanced Institute for Materials Research (AIMR), Tohoku University, 2-1-1 Katahira, Sendai, Miyagi 980-8577 Japan; 2 Graduate School of Mathematics, Nagoya University, Furocho, Nagoya, 464-8602 Japan; 3 Program of Materials Science, School of Engineering, Kwansei Gakuin University, 1 Gakuen-Uegahara, Sanda, Hyogo 669-1337 Japan

**Keywords:** Chemical physics, Metal-organic frameworks

## Abstract

Metal–organic frameworks (MOFs), self-assembled porous materials synthesized from metal ions and organic ligands, are promising candidates for the direct capture of CO_2_ from the atmosphere. In this work, we developed a regression model to predict the optimal component of the MOF that governs the amount of CO_2_ adsorption per volume based on experimentally observed adsorption and structure data combined with MOF adsorption sites. The structural descriptors were generated by topological data analysis with persistence diagrams, an advanced mathematical method for quantifying the rings and cavities within the MOF. This enables us to analyze direct effects and significance of the geometric structure of the MOF on the efficiency of CO_2_ adsorption in a novel way. The proposed approach is proved to be highly correlated with experimental data and thus offers an effective screening tool for MOFs with optimized structures.

## Introduction

Carbon dioxide emissions from modern industrial activities are considered to be the main reason for the greenhouse effect, which causes global warming^1,2^. A certain amount of carbon dioxide is also contained in natural gas whose main component is methane. The presence of carbon dioxide not only reduces the energy efficiency of natural gas but also causes corrosion of pipelines and equipment during natural gas processing. Therefore, the effective capture and separation of carbon dioxide are required to reduce carbon dioxide emissions and improve natural gas quality^3–6^. Currently, chemisorption is employed to remove carbon dioxide from power plant flue gas using an amine solution system. However, amine regeneration requires the thermal cleavage of N–C covalent bonds between carbon dioxide and amine species, resulting in high operating costs^7,8^. Thus, separation by physical adsorption has attracted interest owing to its low cost and high efficiency.

Solid adsorbents that selectively adsorb CO_2_ at partial pressures applicable to CO_2_ capture and storage (CCS) are highly demanded. Metal–organic frameworks (MOFs) formed by the self-assembly of organic ligands and metal ions offer a well-ordered structure suitable for CO_2_ storage owing to their high thermal stability, adjustable chemical functions, and porous features^9^. Various strategies have been developed to enhance the CO_2_ storage capacity of MOFs, including tuning surface properties, such as polar functional groups and open metal sites^10–15^. These methods can significantly increase the CO_2_ adsorption capacity of MOFs owing to the strong interaction between the active sites of MOFs and CO_2_ molecules. However, although numerous MOFs have been synthesized by selecting appropriate organic ligands and metal ions, their optimization for enhanced CO_2_ capture has not yet been demonstrated well. Therefore, it is important to develop a method to efficiently predict the uptake of CO_2_ in MOFs. These predictions have been performed by combining adsorption energies with first-principles calculations and volume/surface area ratios; in addition, machine learning-based approaches have become popular recently^16^. Fernandez et al. introduced the atomic property-weighted radial distribution function (AP-RDF) and reported that the gas adsorption property in the hypothetical MOF (h-MOF) database^17,18^ is well correlated with electronegativity, polarizability, and van der Waals volume at relatively high pressures^19,20^. Anderson et al. developed a machine learning approach based on the Cambridge structural database using descriptors (explanatory variables) involving 16 topologies (crystal lattice types) and 13 functionalized molecular building blocks and reported that the topology of the crystal lattice shows the largest contribution^21^.

Most of the models use simulation-based CO_2_ adsorption data, and interpreting the meaning of their descriptors is not easy to understand. This is the reason why we aim to propose a simple and physically understandable regression/prediction model to evaluate the CO_2_ adsorption using only structural descriptors like the number of adsorption sites and the volume and shape of the adsorption space derived from the structure of MOFs. The key descriptor is by topological data analysis (TDA) based on persistent homology, which is a mathematical framework that enables the quantification of geometric information in the given discrete point data. The births and deaths of the rings (one-dimensional holes) or cavities (two-dimensional holes) are sequentially recorded by increasing the common radius of spheres located at the atomic center, and a two-dimensional histogram that represents the complex structure of a given MOF is obtained.

In this paper, we show that the linear regression (LR) model requires only three explanatory variables to achieve the adjusted coefficient of determination (R2_adj_) of 0.804 for CO_2_ adsorption at 1 bar and 298 K, and the model gives us a clearer understanding of the essential factors compared to other models proposed by data-driven methods only. It is also shown that the shape of the adsorption space quantified by TDA and UMAP improves the model.

## Results

### Preparation of MOF data

It is known that there are three types of adsorption sites that affect the CO_2_ uptake of MOFs: [i] open metal site (OMS), [ii] heteroatom site in a linker, and [iii] the site originating from the secondary building unit (SBU). An OMS is a Lewis acid that accepts the lone pair electrons of the oxygen atom in CO_2_. MOF-74 is known as one of the representative groups^22^. On the other hand, the positively charged center (carbon) in CO_2_ is attacked by Lewis bases. Therefore, aromatic amines or amino groups in MOFs work as chemisorption sites for CO_2_. It has been reported that highly polarized sites such as SiF_6_^23^ or SBU-originated sites called cage window sites^24^ can also have good affinity with CO_2_.

We selected 34 MOFs with various structures, as listed in Table S1 along with their experimentally measured CO_2_ uptake (*wt%*) at 1 bar and 298 K and primary adsorption sites. For some MOFs, the amounts at 1 bar and 298 K were calculated from their isothermal curves. Although CO_2_ uptake is usually reported in *wt%*, we used CO_2_ uptake per unit volume calculated as *wt%/(100 − wt%)* × *density*.

### Description of rings and cavities in MOFs by persistent homology

**Figure 1:**
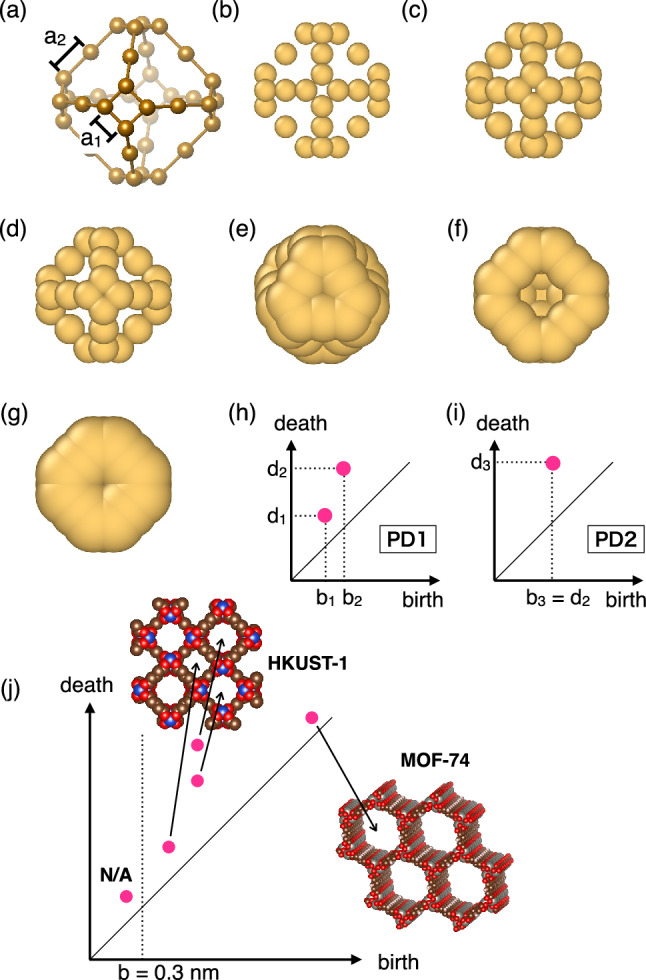
Schematic explanation of the persistence diagram (PD). (**a**) "Atoms" forming a truncated octahedron. (**b**) Births of small rings (*r* *=* *b*_*1*_ *=* *a*_*1*_*/2*). (**c**) Births of large rings (*r* *=* *b*_*2*_ *=* *a*_*2*_*/2*). (**d**) Deaths of small rings (*r* *=* *d*_*1*_). The center of the ring is filled at *r* *=* *d*_*1*_, and the small rings die. (**e**,**f**) The large rings die, and the cavity is born at *r* *=* *d*_*2*_ *=* *b*_*3*_. (**g**) Death of the cavity (*r =* *d*_*3*_). (**h**) First-degree persistence diagram (PD1) of this model cage. (*b*_*1*_, *d*_*1*_) and (*b*_*2*_, *d*_*2*_) represent the small and large rings, respectively. (**i**) Second-degree persistence diagram (PD2) of the model cage. (*b*_*3*_, *d*_*3*_) represents the cavity. (**j**) Characterization of MOFs using PD2.

It is not easy to identify the shape of the complex MOFs visually. A persistence diagram (PD) is a powerful mathematical tool for the TDA of such systems. It is based on the framework of persistent homology, which enables us to record rings and cavities in the given discrete data. A schematic example is as follows: A truncated octahedron with 36 “atoms” is shown in Fig. 1a. By increasing radius *r*, which is common with the spheres at each atomic position, 4-membered small rings and 9-membered large rings are born at *r* = *b*_*1*_ = *a*_*1*_*/2* and *r* = *b*_*2*_ = *a*_*2*_*/2*, respectively (Fig. 1b and c). The 4-membered small rings are filled and die at *r* = *d*_*1*_ (Fig. 1d), and the 9-membered large rings die at *r* = *d*_*2*_ (Fig. 1e). Then, a cavity is born at *r* = *b*_*3*_ = *d*_*2*_ when all the rings are filled and the center space inside the truncated octahedron is separated from the outside. Figure 1f shows the cross section at *r* = *b*_*3*_. The center space is filled at *r* = *d*_*3*_, and the cavity dies (Fig. 1g). Thus, the first-degree persistence diagram (PD1) and the second-degree persistence diagram (PD2) are obtained, as respectively shown in Fig. 1h and i. The rings that have the same shape and size are degenerated into two birth–death pairs on PD1. In addition, the degree of degeneracy at each birth–death pair provides essential information to characterize the system.

Figure 1j shows the characterization of MOFs using PD2. The birth time of the cavity is intuitively interpreted as the largest entrance to the cavity. The death time represents the width inside the cavity. Porous HKUST-1, which is formed by three-dimensionally connected cavities, is characterized by three birth–death pairs. They represent one small and two large cavities. The two large cavities have the same birth time. This shows that they are directly connected to each other. Tubular MOF-74 is characterized by a birth–death pair beside the diagonal line. It has a high degree of degeneracy because each tube is formed by stacking many slices. The entrance to the cavities in MOF-74 is wider than that in HKUST-1. The cavities with birth times less than 0.3 nm are not available for CO_2_ adsorption. 0.3 nm is twice the van der Waals radii of typical atoms in CO_2_ and MOFs.

### Preparation of descriptors for CO_2_ uptake

We focused on three types of structural factors: the number of adsorption sites, the volume fraction of the adsorption space (porosity), and the shape of the adsorption space, which are possible descriptors for CO_2_ uptake and can be derived only from the atomic configurations of MOFs, as shown below (see the Method section for details). Hydrogen atoms are not considered hereafter.

#### Number of adsorption sites

**Figure 2:**
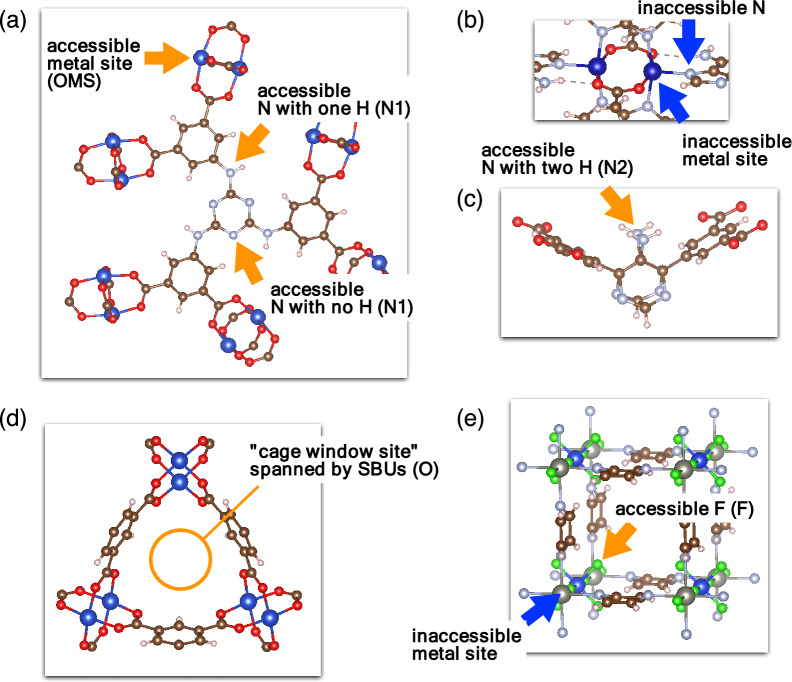
Adsorption sites for CO_2_ in MOFs. (**a**) Accessible open metal site (OMS) and nitrogen sites (N1). (**b**) Examples of inaccessible metal sites and a nitrogen site. (**c**) Accessible nitrogen site originated from the amino group (N2). (**d**) Cage window site (O). (**e**) Accessible fluorine site in the SiF_6_ unit (**F**) and another example of an inaccessible metal site.

We treated five types of adsorption sites: the OMS, nitrogen atom site bonding with less than one hydrogen atom (N1), nitrogen atom site bonding with two hydrogen atoms (N2), triangular space formed by oxygen atoms belonging to the SBU, known as the cage window site (O), and fluorine atom site in the SiF_6_ group (F). Figure 2a shows examples of accessible metal sites and nitrogen sites with one hydrogen, Fig. 2b shows examples of inaccessible metal sites and nitrogen sites, Fig. 2c shows examples of accessible nitrogen sites with two hydrogens, Fig. 2d shows an example of "cage window site" spanning by SBUs, and Fig. 2e shows examples of accessible fluorine and inaccessible metal sites. The variation in metal atom species of OMS may influence the experimentally observed CO_2_ uptake. However, we do not address this aspect here because our focus is on proposing a model using only structural descriptors. The basicity of N1 is weaker than that of N2. O and F are the sites theoretically reported to show large adsorption energies for CO_2_^23–25^. The number of these sites was normalized based on the unit volume of the MOF, but the sites inaccessible for CO_2_ were not counted. In order to represent the contribution from van der Waals interaction based solely on the structural information, the number of nonhydrogen atoms per unit volume (A) was used as a descriptor. It would be a reasonable approximation since MOFs have wireframe structures.

#### Volume fraction of the adsorption space (porosity)

We prepared the volume of cavities in two ways: the volume calculated from PD2 (V_P_) and that evaluated using Zeo++^26^ (V_Z_). The descriptors representing the porosity P and V were obtained by normalizing V_P_ and V_Z_ with the cell volume of the MOF.

#### Shape of the adsorption space

**Figure 3:**
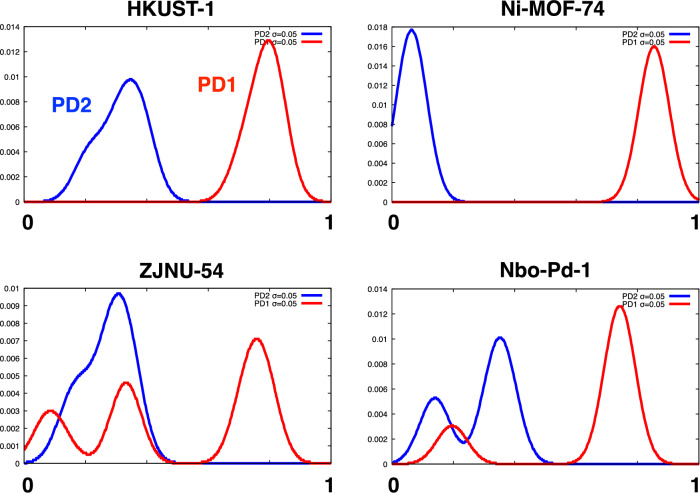
Shapes of rings and cavities in MOFs quantified as the histogram of *(death−birth)/death* values for PD1 (red) and PD2 (blue).

The normalized shapes of the rings or cavities recorded on a PD can be expressed as *(death-birth)/death*. We chose the rings with *death* > *0.3* *nm* or cavities with *birth* > *0.3* *nm* available for CO_2_ adsorption, as mentioned above. Then, the shape of the MOF was characterized by a set of *(death-birth)/death* values for PD1 and PD2. Figure 3 shows four examples of *(death-birth)/death* values with Gaussian blur. By comparing the blue lines in Fig. 3 with PD2 in Fig. 1j, we can see that the shapes of cavities in HKUST-1 and MOF-74 are appropriately represented. ZJNU-54 and Nbo-Pd-1 are also distinguished from HKUST-1 and MOF-74 (their atomic structures are shown in Fig. 5b). Once the *(death-birth)/death* values are expressed as a histogram (or a vector), various machine learning methods can be applied to generate scalar descriptors from the set of vectors for given MOFs.

### Comparison of LR models

We evaluated the contributions of various adsorption sites (OMS, N1, N2, O, F, and A) and porosities (P, Z) to the adsorption of CO_2_. Using the experimentally observed CO_2_ uptake per unit volume at 1 bar and 298 K as an objective variable, LR was performed for various combinations of explanatory variables (descriptors). The results are summarized in Table 1 (see also Table 2S for additional results). Among the LR models with an adjusted coefficient of determination (R2_adj_) greater than 0.8, the simplest one is model MNA/−/− described by only four adsorption sites, OMS, N1, N2, and A. The P-values of OMS, N1, and A are small enough (*< 0.05*) and that of N2 is also acceptable.

**Table 1:** Results of linear regression.

	R2	R2 adj	AIC	BIC	OMS	N1	N2	O	F	A	Vol P, Z	Topo-1	Topo-2
MNA/−/−	0.828	0.804	− 112.32	− 104.69	0.000	0.000	0.105			0.000			
MNAOF/−/−	0.838	0.803	− 110.52	− 99.84	0.000	0.000	0.049	0.458	0.222	0.006			
MNA/P/−	0.849	0.822	− 114.85	− 105.69	0.000	0.000	0.070			0.000	0.056		
MNAOF/P/−	0.857	0.818	− 112.56	− 100.35	0.000	0.000	0.047	(0.865)	0.296	0.001	0.082		
MNOF/P/−	0.787	0.739	− 101.08	− 90.40	0.000	0.000	0.007	0.422	0.027		0.561		
MNA/Z/−	0.831	0.801	− 111.04	− 101.88	0.000	0.000	0.098			0.744	0.446		
MNAOF/Z/−	0.846	0.804	− 110.12	− 97.91	0.000	0.000	0.034	0.296	0.181	(0.458)	(0.273)		
MNOF/Z/−	0.843	0.808	− 111.39	− 100.70	0.000	0.000	0.016	0.384	0.212		(0.004)		
MNA/−/Ss	0.843	0.808	− 111.50	− 100.82	0.000	0.000	0.201			0.001		(0.182)	0.428
MNA/−/Pp	0.852	0.819	− 113.40	− 102.72	0.000	0.000	0.205			0.015		(0.145)	0.389
MNA/−/Uu	0.873	0.845	− 118.82	− 108.14	0.000	0.000	0.267			0.000		0.485	(0.020)
MNA/−/U	0.871	0.848	− 120.20	− 111.04	0.000	0.000	0.260			0.001			0.005

R2: coefficient of determination. R2_adj_: adjusted R2. AIC: Akaike's information criterion. BIC: Bayesian information criterion. P-values are shown for OMS, N1, N2, O, F, A, Vol (P or Z), and Topo-1,2. Topo-1 and Topo-2 represent the first component of the shape characterized by PD1 and PD2, respectively. The way of quantifying Topo-1 and Topo-2 is represented by (p, s, u) and (P, S, U) for PCA, t-SNE, and UMAP. The *P*-value with () represents that the descriptor has a negative coefficient in the linear regression model.

Model MNA/P/− in which PD2-based porosity P is added to model MNA/−/− improves R2_adj_ and AIC/BIC. Here, AIC and BIC are Akaike's information criterion and Bayesian information criterion, which are designed to determine if the combination of descriptors is appropriate. A more negative AIR/BIC is desired for improving the accuracy of the model. Model MNAOF/−/− with additional descriptors O and F also improve R2_adj_, but AIC and BIC values are less negative than those in Model MNA/P/−. The P-values greater than 0.2 for O and F show that these two descriptors are not important in the model, although the reported adsorption energies for O and F sites were sufficiently large, 25 kJ/mol^25^ and 33 kJ/mol^23^, respectively. The adsorption energy for the OMS was reported as 15 kJ/mol^25^. Regarding F sites, it may be because only three of 34 MOFs comprise F sites. However, the number of MOFs with O sites is comparable to that with OMS sites.

Model MNAOF/P/− improves R2_adj_ and AIC, but BIC is degraded. A more serious problem is that the coefficient for O in the LR model is negative, although the number of adsorption sites is expected to show a positive contribution to CO_2_ adsorption. In addition, O is not a suitable descriptor in this model. Comparing model MNOF/P/− with model MNAOF/P/−, we found that the removal of A remarkably degrades R2_adj_, AIC, and BIC, implying that A is an essential descriptor for the evaluation of CO_2_ uptake. The large P-value of P implies that P is not always necessary.

The comparison among models MNA/−/−, MNA/Z/−, and MNAOF/Z/− shows that the P-value of A becomes significantly large upon the addition of porosity Z obtained using Zeo+ +. Further, upon comparison with model MNAOF/−/−, we deduced that the P-value of Z in model MNOF/Z/− is less than 0.01 and that Z may be substituted for A.

**Figure 4:**
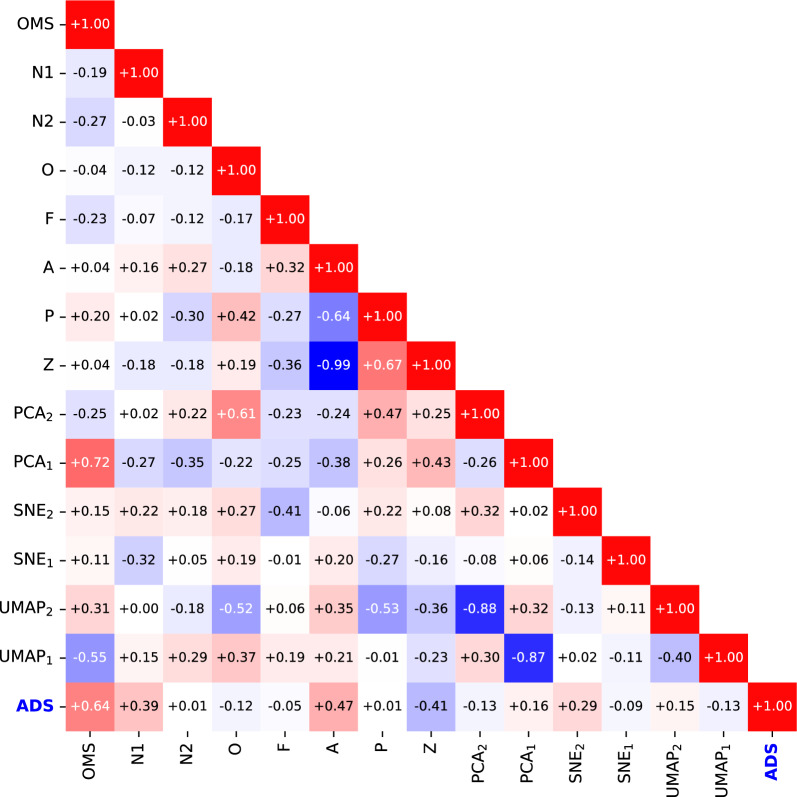
Correlation map of descriptors. ADS is the objective variable (CO_2_ uptake per unit volume). The color denotes the heat map (red: positive correlation, blue: negative correlation).

The correlation map of descriptors and ADS (CO_2_ uptake per unit volume) gives a perspective on the results above (Fig. 4). The good performance of model MNA/−/− is intuitively understood by the fact that OMS, A, and N1 show a positive correlation with ADS. N2 has no correlation with ADS despite its relatively small P-value of 0.1. This is likely because only three of 34 MOFs have nonzero N2.

The descriptors Z and A show a completely negative correlation of − 0.99. Correlations with the other descriptors show a clearly opposite trend between A and Z. As the P values of A and Z are greater than 0.2 in models MNA/Z/− and MNAOF/Z/−, A and Z are considered as the same descriptor because the total MOF volume is the summation of the volume of cavity (V_Z_) and the van der Waals volume proportional to A.

The descriptors P and A also show a large negative correlation of − 0.64. P and A show, however, different trends of correlations, especially for the shape of the adsorption space Topo-1 and Topo-2 (PCA_1_, PCA_2_, SNE_1_, SNE_2_, UMAP_1_, and UMAP_2_: see the next section). Since P originates from the PD2-based volumes with the condition of *b* > *0.3* *nm*, its structural information is different from that of Z and A. Therefore, P does not conflict with A in models MNA/P/− and MNAOF/P/−.

### Contribution from the shape of the adsorption space

The shape of the adsorption space can also contribute to the improvement of the regression model. Information on the shape is summarized as PD1 and PD2, but generating descriptors from them is not trivial. In this paper, we converted PD1 and PD2 into 500-dimensional vectors, which represent the distribution of *(death-birth)/death*. These vectors can be quantified as a set of scalar values using a dimensionality reduction method, such as PCA, t-SNE, and UMAP. While PCA is based on linear algebra, t-SNE and UMAP are based on nonlinear analysis, such as statistical geometry and/or manifold learning theory. t-SNE defines a lower dimensional space by minimizing the probability distribution of the entire data in the higher dimensional space, whereas UMAP assumes that a Riemannian manifold (higher dimensional curved space) on which entire data is distributed defines a lower dimensional space by minimizing cross entropy between the data projected on a lower dimensional space and the entire data.

The descriptors (scalar values) obtained by PCA, t-SNE, and UMAP are named p, s, and u for PD1 and P, S, and U for PD2, respectively. For example, model MNA/P/pPP expresses model MNA/−/− extended by the PD2-based volume (P), the first component of PCA on PD1 (p), and the first and second components of PCA on PD2 (PP). Topo-1 or Topo-2 in Table 1 represents the first component of "p, s, and u" or "P, S, and U".

**Figure 5:**
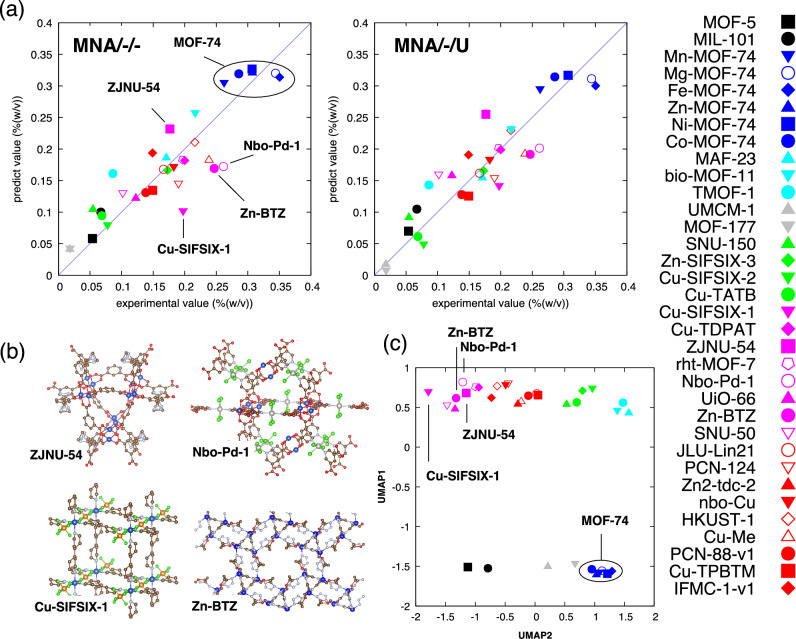
Contribution from the shape of the adsorption space. (**a**) Comparison of the regression results for each MOF between model MNA/−/− and model MNA/−/U. The horizontal and vertical axes show the experimental and predicted values, respectively. The prediction of Cu-SIFSIX-1, Zn-BTZ, and Nbo-Pd-1 is clearly improved, while that of ZJNU-54 is degraded. (**b**) Atomic structures of Cu-SIFSIX-1, Zn-BTZ, Nbo-Pd-1, and ZJNU-54. (**c**) Distribution of MOFs in a feature value space with UMAP1 and UMAP2. The color of the symbols is assigned based on this map to see the trend of correction by the shape of the adsorption space.

As shown in Table 1 and Table S2, UMAP-based descriptors improve model MNA/−/− more effectively than PCA and t-SNE. In particular, model MNA/−/U shows the best combination of R2_adj_ , AIC, and BIC. The detailed regression results of models MNA/−/− and MNA/−/U are visualized in Fig. 5a. The horizontal axis shows the measurement values, and the vertical axis shows the predicted values, respectively. From the comparison of models MNA/−/− and MNA/−/U, we can deduce that the prediction of some MOFs such as Cu-SIFSIX-1, Zn-BTZ, and Nbo-Pd-1 is clearly improved, whereas that of others such as ZJNU-54 is degraded. Figure 5b shows four examples of atomic models of such MOFs, but it is hard to find their structural similarities or differences with the naked eye. Figure 5c shows a two-dimensional map obtained using the first components of UMAP on PD1 and PD2 (UMAP1 and UMAP2). While UMAP2 shows a continuous distribution, the characterization by UMAP1 is discrete. The color of the symbols is assigned based on this map to see the trend of correction. The MOFs with negative UMAP2 show positive corrections of predicted values, and those with positive UMAP2 show negative corrections. It is difficult to obtain an intuitive explanation about which structural feature is quantified by UMAP2, but we can expect that the shape of the cavity also affects CO_2_ uptake.

### Proposed linear regression models

Based on the results and consideration above, model MNA/−/− using four descriptors (OMS, N1, N2, and A) is proposed as the simplest and practical LR model for CO_2_ uptake at 1 bar and 298 K. This is given as

$${\text{ADS}}_{{{\text{MNA}}/ - / - }} = { 4}.{\text{742 X}}_{{{\text{OMS}}}} + {2}.{\text{794 X}}_{{{\text{N1}}}} + {1}.{\text{169 X}}_{{{\text{N2}}}} - \, 0.{\text{268 X}}_{{\text{A}}} + {16}.{153}$$

using raw descriptors or

$${\text{ADS}}_{{{\text{MNA}}/ - / - }} = \, 0.0{\text{742 X}}_{{{\text{OMS}},{\text{std}}}} + 0.0{\text{474 X}}_{{{\text{N1}},{\text{std}}}} + 0.0{\text{138 X}}_{{{\text{N2}},{\text{std}}}} + 0.0{\text{321 X}}_{{{\text{A}},{\text{std}}}} + 0.{1824}$$

using standardized descriptors to compare the contribution of each component.

Model MNA/−/U is proposed as the simplest model with additional information on the shape derived by TDA and UMAP. It consists of OMS, N1, N2, A, and U (the 1st component of UMAP on PD2) and given as

$${\text{ADS}}_{{{\text{MNA}}/ - /{\text{U}}}} = { 5}.{\text{273 X}}_{{{\text{OMS}}}} + {2}.{\text{744 X}}_{{{\text{N1}}}} + 0.{\text{653 X}}_{{{\text{N2}}}} + 0.{\text{661 X}}_{{\text{A}}} - {2}.{\text{315 X}}_{{\text{U}}} + {16}.{153}$$

using raw descriptors or

$${\text{ADS}}_{{{\text{MNA}}/ - /{\text{U}}}} = \, 0.0{\text{796 X}}_{{{\text{OMS}},{\text{std}}}} + 0.0{\text{468 X}}_{{{\text{N1}},{\text{std}}}} + 0.00{\text{86 X}}_{{{\text{N2}},{\text{std}}}} + 0.0{\text{416 X}}_{{{\text{A}},{\text{std}}}} - \, 0.0{\text{236 X}}_{{{\text{U}},{\text{std}}}} + 0.{1824}$$

using standardized variables to compare the contribution of each component.

OMS, N1, and N2 are the primary adsorption sites for CO_2_ in MOFs according to Yaghi et al.^27^ (see Table S1). On the other hand, our models show that the contributions from OMS, N1, and A are dominant. A is the descriptor proportional to the number of atoms in the MOF, and we introduced it to improve the prediction of CO_2_ uptake for the MOFs with no OMS, N1, and N2 sites (UMCM-1, MOF-177, SNU-150, TMOF-1, and MOF-5). However, it appears that A has a strong negative correlation with porosity Z obtained using Zeo++, as shown in Fig. 4. Does A work as a van der Waals adsorption site or just as an adsorption space? Considering that the density of CO_2_ in free space at 1 bar and 298 K is 1.8 × 10^−3^ g/cm^3^, which is smaller than the CO_2_ uptake of UMCM-1 (1.8 × 10^−2^ g/cm^3^), we can conclude that the descriptor A works as a van der Waals adsorption site.

## Discussion

We showed a physically and chemically understandable, simple regression/prediction model can be constructed using only geometric descriptors that capture the structural features of MOFs and proposed a framework to achieve it by PD based TDA of the atomic configurations of MOFs.

Using the experimentally obtained data for 34 MOFs at 1 bar and 298 K, we found that the CO_2_ uptake per volume can be predicted based on a simple LR model (model MNA/−/−, R2_adj_ = 0.80) using only the numbers of OMS, N1, N2, and all nonhydrogen atoms (A). It gives a clear interpretation of the descriptors (polarizability, electronegativity, and van der Waals volume) used in the model by Woo et al.^19,20^. The descriptors for the porosity (P, Z) of MOFs did not contribute to CO_2_ uptake at 1 bar and 298 K. In addition, the shapes of cavities (U) quantified by TDA and UMAP improved the performance of regression (model MNA/-/U, R2_adj_ = 0.85).

However, outliers remain in the regression results of model MNA/−/− (Fig. 5a). For example, while Cu-SIFSIX-2 and Zn-SIFSIX-3 predict the observed values well, Cu-SIFSIX-1 gives a significant error of *−* *44%*. SIFSIX is a family of MOFs with a jungle gym shape and the same number of SiF_6_ units in a cell, and the size of Cu-SIFSIX-1 is the middle of Cu-SIFSIX-3 and Zn-SIFSIX-3. On the other hand, the prediction value of Cu-SIFSIX-1 in model MNA/−/U is much improved (error: *−* *24%*). According to the report based on DFT calculations^23^, Cu-SIFSIX-1 has three different structural isomers with different CO_2_ uptake values. Considering that they have no OMS, N1, and N2 sites, it is surprising that a good prediction of Cu-SIFSIX-3 and Zn-SIFSIX-3 is obtained using only A. It is also reasonable that the prediction of Cu-SIFSIX-1 fails without considering its shape. The outliers whose regression results substantially change between models MNA/−/− and MNA/−/U show that the effects of isomers (structural dynamics) on the amount of CO_2_ adsorption must also be identified.

Morozov et al. proposed a framework for the estimation of gas uptake by combining TDA-based structural data and word embedding-based chemical composition data. They analyzed the h-MOF and CoreMOF database^28,29^ and reported that TDA is a major descriptor for the prediction of CH_4_ uptake, but the word embedding-based descriptor becomes major for CO_2_ uptake. Although their results are similar to those presented in this work, the generation and interpretation of word embedding-based descriptors are not easy. Therefore, we focused on the construction of simple and practical models with a small number of descriptors for the MOFs whose CO_2_ uptake was experimentally studied and obtained encouraging linear regression results achieving the adjusted coefficient of determination (R2_adj_) of 0.804. Predictability of the models were also confirmed by cross validation. (Fig. S3). This approach provides a novel usage of TDA. It is easy to modify the structural descriptors in our linear models using the coefficients given as functions of temperature and/or pressure keeping the physical picture clear. We will further perform the screening of the CoreMOF database and its experimental verification.

## Methods

### Preparation of atomic configurations

The atomic configurations in the XYZ format were generated from the CIF data using the "cif2cell" tool. The atoms from isolated or coordinated solvent fragments were removed. The atoms whose occupancy was less than 1 were also partially removed considering the bond length, bond angle, and coordination number. The obtained atomic configurations were converted to CIF files in the P1 space group.

### Calculation of persistent homology

The persistent homology of the given point cloud data was calculated using open-source software “HomCloud”^30^. For the input XYZ data, a set of birth–death pairs and corresponding birth and death simplices were obtained for the zeroth-, first-, and second-degree persistence diagrams (PD0, PD1, and PD2). The death simplex for PD2 is a tetrahedron representing the cavity. PH tree was also calculated to obtain the whole simplices (or atoms) forming the cavity.

### Counting chemical adsorption sites

Each atomic configuration was prepared as a 3 × 3 × 3 supercell, and the adsorption sites were extracted as local geometric structures from the 1 × 1 × 1 supercell at the center as follows:

#### OMS

For each metal atom in the center 1 × 1 × 1 cell, its local configuration was generated by extracting atoms within a sphere of radius 0.5 nm. The birth–death pairs in PD2 of the local configurations with/without the center atom were compared to extract the active open metal sites (OMS). Specifically, the sites which meet the following conditions were classified as "inactive" metal sites: (1) there is no cavity including the center atom in its vertices and removal of the central atom generates a cavity whose lifetime (*death* − *birth*) is larger than 0.004 nm, or (2) there is at least one cavity including the center atom in its vertices and removal of the central atom generates a cavity whose lifetime is larger than 0.01 nm (See also Fig. S1 showing typical examples).

#### Nitrogen sites (N1, N2)

Accessible nitrogen atoms were extracted, as performed in the case of an OMS, and classified into N1 (bonding with less than one hydrogen atom) and N2 (bonding with two hydrogen atoms).

#### Oxygen sites (O)

A point cloud data comprising oxygen atoms only was prepared. The nearly cubic SBU formed by eight oxygen atoms was detected using PD2, as shown in Fig. 2d. A new point cloud data with the center of mass of the SBU was prepared and its PD1 was calculated. The number of oxygen sites was obtained by counting nearly regular triangles whose death/birth ratio is $2/\sqrt{3}$. Figure S2 schematically shows the procedure.

#### Fluorine sites (F)

The number of accessible fluorine atoms was counted, as in the case of an OMS.

### Evaluation of the volume of the adsorption space (porosity)

#### PD2-based porosity (P)

The volume of the tetrahedrons, which comprised a polygon of PH tree, was added, and the following properties were satisfied:

- Their death time was greater than 0.3 nm.
- The center of their circumsphere was contained in the 2 × 2 × 2 supercell.
- They were not a member of a subpolygon.

Then, normalize it by the volume of the supercell.

#### Zeo++-based porosity (Z)

The volume was estimated by 50000 random trials of accessibility of the probe using open-source utility Zeo++^26^ such as “typewriter face.” Then, it was normalized by the volume of the supercell.

### Fingerprints of the shape of the MOF based on TDA

We selected the birth–death pairs on PD1 (PD2) with a center of the circumcircle (circumsphere) for the death triangle (tetrahedron) in the 2 × 2 × 2 supercell. We considered only the rings with *death* > *0.3* *nm* and cavities with *birth* > *0.3* *nm* were active for CO_2_ adsorption. Then, the set of "*1* b/d" was summarized as a histogram in increments of 0.01, normalized by the number of birth–death pairs. Finally, we applied Gaussian blur with σ = 0.01 and obtained the fingerprints (vectors) representing the probability density of the normalized shape for the given MOF.

### Quantification of the fingerprints of the shape by t-SNE and UMAP

The results of t-SNE and UMAP varied depending on the dimension *n* to be mapped. Figure 5c shows the results obtained by UMAP with *n* = *3*. The regression results in Table S2 in SI were obtained by t-SNE and UMAP with *n* = *3*, even if the first coefficients of t-SNE or UMAP were used.

### Supplementary Information

Supplementary Information.

## Data Availability

The data that support the findings of this study are available from the corresponding author upon reasonable request.
