# Supplementary material for: Linear regression model for metal–organic frameworks with CO2 adsorption based on topological data analysis
Source: Sci Rep. 2024 May 26;14:12021. doi: 10.1038/s41598-024-62858-7 (PMC11128442; doi:10.1038/s41598-024-62858-7)
Supplement: Supplementary file 1 — Supplementary Information. [file 41598_2024_62858_MOESM1_ESM.pdf]

Linear regression model for metal organic frameworks with CO_2 adsorption based on topological data analysis

Kazuto Akagi*[1], Hisashi Naito[2], Takafumi Saikawa[2], Motoko Kotani[1], Hirofumi Yoshikawa[3]

1 Advanced Institute for Materials Research (AIMR), Tohoku University, 2-1-1 Katahira, Sendai, Miyagi 980-8577, Japan

2 Graduate School of Mathematics, Nagoya University, Nagoya, 464-8602, Japan

3 Program of Materials Science, School of Engineering, Kwansei Gakuin University, 1 Gakuen-Uegahara, Sanda, Hyogo 669-1337, Japan

Materials	CO_2 Uptake (wt%)	Primary adsorption site	Year	DOI
UMCM-1	4.5		2020	10.1016/j.micromeso.2019.109844
MOF-177	4.7	-	2021	10.1016/j.jece.2021.105523
SNU-150	6.1		2013	10.1002/chem.201303086
TMOF-1	6.2		2016	10.1021/acs.chemmater.6b02511
MOF-5	8.5		2009	10.1021/ie900665f
UiO-66	9.1		2015	10.1016/j.jiec.2014.12.021
Zn-SIFSIX-3	10.0	SBU-based interactions	2013	10.1038/nature11893
Cu-SIFSIX-2	10.8	SBU-based interactions	2012	10.1021/ja211340t
MAF-23	11.0		2012	10.1021/ja3073512
IFMC-1	11.9		2012	10.1039/C2SC00017B
MIL-101	13.7	OMS	2011	10.1021/ef101548g
bio-MOF-11	15.3	Amines	2010	10.1021/ja909169x
Cu-TATB	15.6	-	2011	10.1039/C0JM03318A
SNU-50	16.0		2010	10.1002/chem.201002135
Zn(BTZ) (alias: Zn-BTZ)	18.0	Heteroatom	2012	10.1021/ja3063138
PCN-124	18.4	Hybrid	2016	10.1002/chem.201504907
PCN-88	18.5		2013	10.1038/ncomms2552
JLU-Liu21	18.8	Hybrid	2016	10.1039/C5CC09922F
ZJNU-54	19.1	Heteroaromatic amine	2016	10.1039/C6DT02150F
Cu-SIFSIX-1	19.1	Heteroaromatic amine	2012	10.1021/ja211340t
nbo(Cu2(DBIP)(H2O)2) (alias: nbo-Cu)	19.3	OMS	2013	10.1021/ic4017189
Cu-TPBTM	19.5		2011	10.1021/ja110042b
Co-MOF-74	19.7	OMS	2009	10.1021/ja9057234
NbO-Pd-1	19.7	Hybrid	2016	10.1039/C6CC04790D
HKUST-1	19.8	OMS	2010	10.1021/je1002225
Zn-MOF-74	20.1	OMS	2013	10.1039/C3SC51319J
rht-MOF-7	20.2	Heteroaromatic amine	2014	10.1021/cg401802s
Ni-MOF-74	20.5	OMS	2009	10.1021/ja9057234
Cu-TDPAT	20.6	Hybrid	2012	10.1002/anie.201105966
Cu(Me-4py-trz-ia) (alias: Cu-Me)	21.1	OMS	2014	10.1039/C4CC03070B
Mn-MOF-74	22.5	OMS	2013	10.1039/C3SC51319J
Fe-MOF-74	23.8	OMS	2012	10.1016/j.micromeso.2011.12.035
Zn2(tdc)2(MA) (alias: Zn2-tdc-2)	27.0	OMS	2016	10.1016/j.molstruc.2015.11.045
Mg-MOF-74	27.5	OMS	2011	10.1016/j.jcis.2010.09.065

Table S1. Summary of the references of the MOFs used in this paper. "alias" means the abbreviated name shown in the main text and Table S3.

Model	Adj R2	AIC	BIC	OMS	N1	N2	O	F	A	"Vol (P, Z)"	T11	T12	T21	T22
MNAOF/-/Uu	0.854	-119.335	-105.598	0.000	0.000	0.445	0.141	0.582	0.000	-	0.221	-	0.008	-
MNAOF/-/U	0.850	-119.258	-107.047	0.000	0.000	0.341	0.274	0.486	0.000	-	-	-	0.005	-
MNAO/P/U	0.850	-119.082	-106.871	0.000	0.000	0.410	0.151	-	0.000	0.552	-	-	0.014	-
MNAOF/P/UU	0.849	-117.724	-102.461	0.000	0.000	0.182	0.428	0.568	0.000	0.408	-	-	0.239	-
MNAOF/P/Uu	0.848	-117.524	-102.260	0.000	0.000	0.420	0.140	0.588	0.002	0.718	0.264	-	0.026	-
MNA/-/U	0.848	-120.196	-111.038	0.000	0.000	0.260	-	-	0.000	-	-	-	0.005	-
MNAOF/P/PPpp	0.848	-116.329	-98.012	0.000	0.000	0.503	0.037	0.863	0.066	0.225	0.020	0.035	0.440	0.754
MNAOF/P/U	0.847	-117.719	-103.981	0.000	0.000	0.307	0.245	0.498	0.000	0.564	-	-	0.023	-
MNAF/-/Uu	0.846	-118.321	-106.110	0.000	0.000	0.157	-	0.289	0.001	-	0.482	-	0.023	-
MNA/-/Uu	0.845	-118.821	-108.137	0.000	0.000	0.267	-	-	0.000	-	0.485	-	0.020	-
MNAF/P/U	0.844	-117.846	-105.635	0.000	0.000	0.146	-	0.287	0.001	0.711	-	-	0.046	-
MNAOF/-/PPpp	0.844	-116.000	-99.210	0.000	0.000	0.605	0.047	0.963	0.156	-	0.016	0.046	0.145	0.543
MNA/P/U	0.843	-118.334	-107.649	0.000	0.000	0.249	-	-	0.000	0.743	-	-	0.039	-
MNAOF/-/UUuu	0.840	-115.143	-98.353	0.000	0.000	0.578	0.093	0.904	0.021	-	0.021	0.266	0.421	0.092
MNAOF/P/UUuu	0.835	-113.605	-95.288	0.000	0.000	0.492	0.106	0.910	0.031	0.589	0.034	0.263	0.408	0.147
MNAOF/P/Pp	0.828	-113.316	-98.052	0.000	0.000	0.224	0.224	0.292	0.044	0.312	0.104	-	0.321	-
MNA/-/UUuu	0.828	-113.863	-100.126	0.000	0.000	0.172	-	-	0.015	-	0.046	0.366	0.339	0.207
MNAOF/-/Pp	0.828	-113.838	-100.101	0.000	0.000	0.286	0.249	0.229	0.072	-	0.072	-	0.120	-
MNAF/-/UUuu	0.826	-112.870	-97.606	0.000	0.000	0.124	-	0.404	0.025	-	0.094	0.425	0.342	0.186
MNAF/-/Pp	0.825	-113.991	-101.780	0.000	0.000	0.098	-	0.163	0.054	-	0.144	-	0.273	-
MNA/-/PPpp	0.825	-113.161	-99.424	0.000	0.000	0.161	-	-	0.051	-	0.091	0.103	0.769	0.701
MNAF/P/S	0.824	-113.675	-101.464	0.000	0.000	0.067	-	0.178	0.001	0.085	-	-	0.352	-
MNAOF/P/SS	0.823	-112.155	-96.892	0.000	0.000	0.154	0.484	0.203	0.001	0.160	-	-	0.218	-
MNA/P/-	0.822	-114.851	-105.693	0.000	0.000	0.070	-	-	0.000	0.056	-	-	-	-
MNAF/-/PPpp	0.822	-112.064	-96.801	0.000	0.000	0.119	-	0.429	0.084	-	0.112	0.243	0.602	0.710
MNAF/P/P	0.820	-113.040	-100.829	0.000	0.000	0.066	-	0.218	0.002	0.233	-	-	0.533	-
MNA/-/p	0.820	-114.448	-105.290	0.000	0.000	0.130	-	-	0.021	-	0.068	-	-	-
MNAF/-/UU	0.819	-112.805	-100.595	0.000	0.000	0.121	-	0.176	0.001	-	-	-	0.462	0.131
MNAF/-/SS	0.819	-112.776	-100.565	0.000	0.000	0.102	-	0.201	0.001	-	-	-	0.056	0.860
MNA/-/Pp	0.819	-113.401	-102.716	0.000	0.000	0.205	-	-	0.015	-	0.145	-	0.389	-
MNAOF/P/S	0.818	-111.960	-98.223	0.000	0.000	0.099	0.650	0.217	0.001	0.087	-	-	0.315	-
MNAOF/P/-	0.818	-112.561	-100.350	0.000	0.000	0.047	0.865	0.296	0.001	0.082	-	-	-	-
MNA/P/S	0.818	-113.257	-102.572	0.000	0.000	0.129	-	-	0.000	0.076	-	-	0.574	-
MNA/-/u	0.817	-113.867	-104.709	0.000	0.000	0.134	-	-	0.001	-	0.090	-	-	-
MNA/P/P	0.817	-113.017	-102.333	0.000	0.000	0.119	-	-	0.000	0.177	-	-	0.719	-
MNAOF/P/P	0.816	-111.499	-97.762	0.000	0.000	0.126	0.565	0.266	0.002	0.213	-	-	0.411	-
MNAOF/P/Ss	0.816	-110.862	-95.598	0.000	0.000	0.099	0.890	0.256	0.001	0.161	0.430	-	0.400	-
MNA/-/SS	0.814	-112.600	-101.916	0.000	0.000	0.190	-	-	0.000	-	-	-	0.068	0.587
MNAO/P/S	0.814	-111.849	-99.638	0.000	0.000	0.180	0.505	-	0.000	0.064	-	-	0.455	-
MNAO/P/P	0.814	-111.785	-99.574	0.000	0.000	0.213	0.448	-	0.000	0.156	-	-	0.475	-
MNAOF/-/UU	0.813	-111.064	-97.326	0.000	0.000	0.222	0.666	0.221	0.002	-	-	-	0.512	0.123
MNA/-/UU	0.813	-112.362	-101.677	0.000	0.000	0.243	-	-	0.000	-	-	-	0.505	0.168
MNAF/-/PP	0.812	-111.468	-99.258	0.000	0.000	0.099	-	0.168	0.002	-	-	-	0.113	0.620
MNAOF/-/SS	0.812	-110.783	-97.046	0.000	0.000	0.124	0.945	0.210	0.002	-	-	-	0.081	0.854
MNAF/-/Ss	0.812	-111.416	-99.205	0.000	0.000	0.117	-	0.231	0.002	-	0.258	-	0.268	-
MNAOF/-/P	0.811	-111.344	-99.133	0.000	0.000	0.173	0.696	0.194	0.002	-	-	-	0.146	-
MNA/-/s	0.811	-112.694	-103.536	0.000	0.000	0.107	-	-	0.000	-	0.166	-	-	-
MNA/-/P	0.810	-112.676	-103.518	0.000	0.000	0.210	-	-	0.000	-	-	-	0.168	-
MNA/-/Ss	0.808	-111.501	-100.816	0.000	0.000	0.201	-	-	0.000	-	0.182	-	0.428	-
MNAOF/P/PP	0.808	-109.499	-94.235	0.000	0.000	0.134	0.573	0.276	0.002	0.222	-	-	1.000	-
MNAOF/-/Ss	0.808	-110.026	-96.288	0.000	0.000	0.097	0.507	0.216	0.002	-	0.214	-	0.431	-
MNOF/Z/-	0.808	-111.389	-100.704	0.000	0.000	0.016	0.384	0.212	-	0.004	-	-	-	-
MNAOF/-/PP	0.806	-109.743	-96.006	0.000	0.000	0.173	0.657	0.199	0.003	-	-	-	0.143	0.592
MNAF/-/-	0.806	-111.816	-102.658	0.000	0.000	0.058	-	0.272	0.005	-	-	-	-	-
MNAF/-/SSss	0.806	-109.044	-93.780	0.000	0.000	0.114	-	0.275	0.002	-	0.673	0.849	0.070	0.793
MNA/-/PP	0.805	-110.939	-100.255	0.000	0.000	0.205	-	-	0.000	-	-	-	0.176	0.650
MNAOF/P/SSss	0.805	-107.898	-89.581	0.000	0.000	0.111	0.853	0.391	0.001	0.185	0.750	0.910	0.175	0.621
MNAOF/Z/-	0.804	-110.123	-97.913	0.000	0.000	0.034	0.296	0.181	0.458	0.273	-	-	-	-
MNA/-/-	0.804	-112.322	-104.690	0.000	0.000	0.105	-	-	0.000	-	-	-	-	-
MNA/-/SSss	0.804	-109.320	-95.583	0.000	0.000	0.184	-	-	0.001	-	0.561	0.835	0.089	0.577
MNAOF/-/S	0.803	-109.888	-97.677	0.000	0.000	0.104	0.718	0.160	0.005	-	-	-	0.312	-
MNAOF/-/-	0.803	-110.523	-99.838	0.000	0.000	0.049	0.458	0.222	0.006	-	-	-	-	-
MNA/-/S	0.802	-111.220	-102.062	0.000	0.000	0.204	-	-	0.000	-	-	-	0.394	-
MNA/Z/-	0.801	-111.040	-101.882	0.000	0.000	0.098	-	-	0.744	0.446	-	-	-	-
MNAOF/-/SSss	0.798	-107.118	-90.328	0.000	0.000	0.129	0.824	0.276	0.003	-	0.646	0.800	0.085	0.736
MNAOF/V/-	0.795	-108.526	-96.315	0.000	0.000	0.057	0.485	0.231	0.015	0.963	-	-	-	-
MNOF/V/-	0.751	-102.632	-91.948	0.000	0.000	0.019	0.375	0.033	-	0.214	-	-	-	-
MNOF/P/-	0.739	-101.084	-90.400	0.000	0.000	0.007	0.422	0.027	-	0.561	-	-	-	-
-/Z/-	0.145	-64.936	-61.884	-	-	-	-	-	-	0.015	-	-	-	-
-/V/-	-0.004	-59.457	-56.404	-	-	-	-	-	-	0.360	-	-	-	-
-/P/-	-0.031	-58.556	-55.503	-	-	-	-	-	-	0.955	-	-	-	-

Table S2. Summary of the linear regression for various models. R2: coefficient of determination. adj R2: adjusted R2. AIC: Akaike's information criterion. BIC: Bayesian information criterion. P-values are shown for OMS, N1, N2, O, F, A, Vol, and Topo-1,2.

(a) not OMS

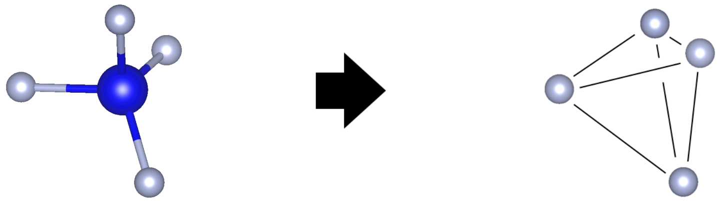

no cavity (birth, death) = (1.93, 1,99)

(b) not OMS

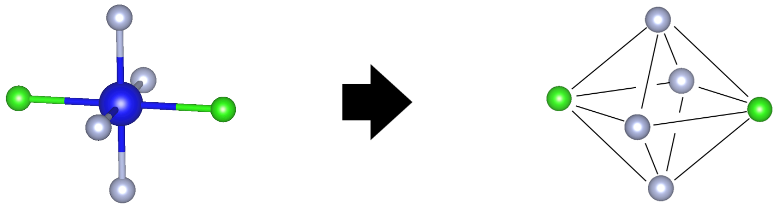

no cavity (1.75, 2.04)

(c) not OMS

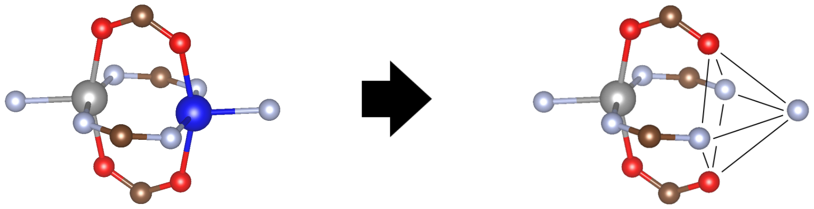

(1,74, 1.78), (1.74, 1.78) (1.84, 2.06)

(d) OMS

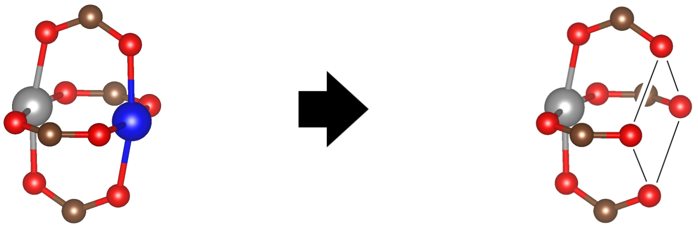

(1,74, 1.78), (1.74, 1.78) (1.95, 2.00)

(e) OMS

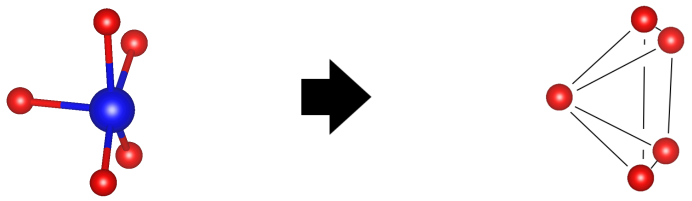

no cavity (2.00, 2.01) → no cavity

Figure S1. Typical examples of metal sites and their classification. Each blue atom is the candidate of OMS. The essential cavities in PD2 are expressed as (birth, death). Examples (a) and (b) have no cavity including the blue atom and removal of it generates a cavity whose lifetime (deathbirth) is larger than 0.004 nm. Example (c) has two cavities, one of whose vertices is the blue atom and removal of it generates a large cavity whose lifetime is more than 0.01 nm. Hence (a), (b), and (c) are not OMS, and the others (d) and (e) are classified as OMS.

(a) (b)

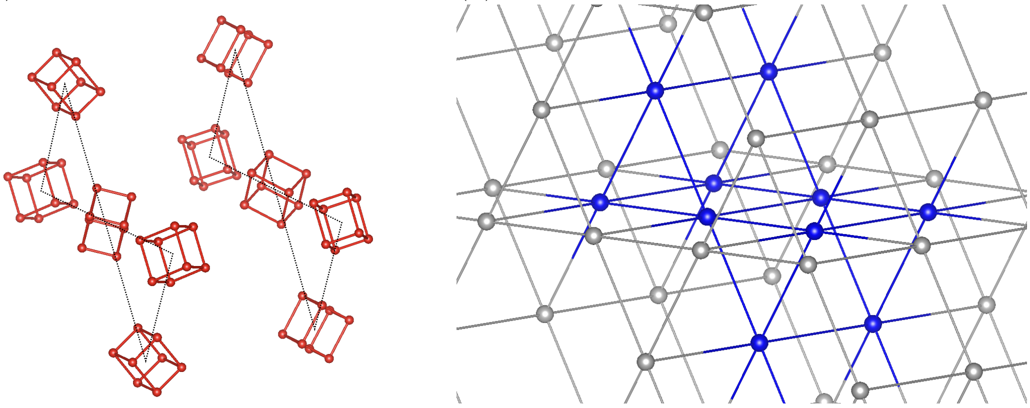

Figure S2. Schematic figures for identification of the oxygen sites. (a) cubic building unit formed by eight oxygen atoms, which are detected by PD2. (b) oxygen sites, which form nearly regular triangles whose death/birth ratio is $2/\sqrt{3}$. The cubes in (a) are corresponding to the blue spheres in (b).

(a)

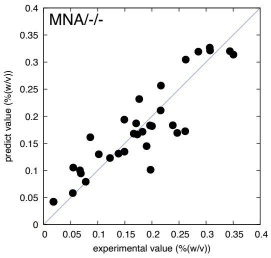

	adj R2	AIC	BIC
(a)	+0.804	−112.3	−104.7
(b)	+0.707	−98.7	−91.1
(c)	+0.796	−111.0	−103.3
(d)	+0.566	−85.4	−77.8

(b)

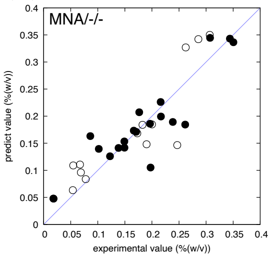

(c)

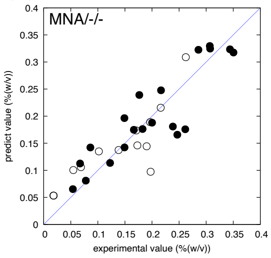

(d)

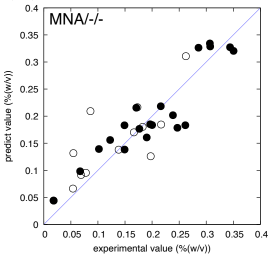

(e)

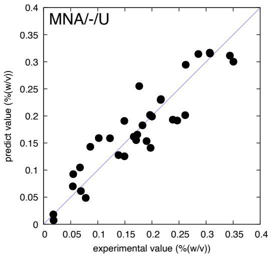

	adj R2	AIC	BIC
(e)	+0.848	−120.2	−111.1
(f)	+0.771	−106.2	−97.1
(g)	+0.843	−119.0	−109.9
(h)	+0.462	−77.2	−68.0

(f)

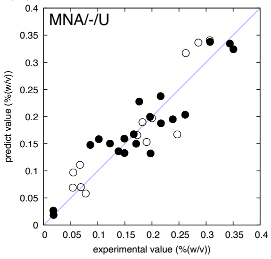

(g)

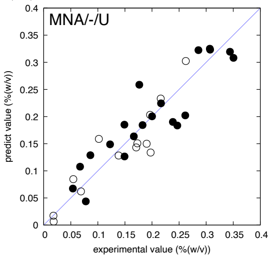

(h)

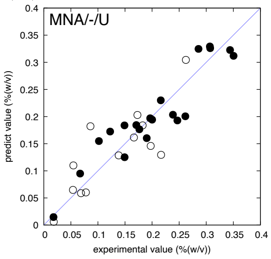

(i)

	G1	G2	G3
MOF-5	P	L	P
MIL-101	P	L	L
Mn-MOF-74	P	P	P
Mg-MOF-74	L	L	L
Fe-MOF-74	L	L	L
Zn-MOF-74	L	L	L
Ni-MOF-74	P	L	L
Co-MOF-74	P	L	L
MAF-23	L	P	L
bio-MOF-11	L	L	P
TMOF-1	L	L	P
UMCM-1	L	P	L
MOF-177	L	P	P
SNU-150	P	P	P
Zn-SIFSIX-3	P	P	P
Cu-SIFSIX-2	P	L	P
Cu-TATB	P	P	P
Cu-SIFSIX-1	L	P	P
Cu-TDPAT	P	L	L
ZJNU-54	L	L	L
rht-MOF-7	L	P	L
Nbo-Pd-1	L	L	L
UiO-66	L	L	L
Zn-BTZ	P	L	L
SNU-50	L	P	L
JLU-Lin21	L	L	P
PCN-124	P	P	L
Zn2-tdc-2	P	L	P
nbo-Cu	P	L	P
HKUST-1	L	P	L
Cu-Me	L	L	L
PCN-88	L	P	P
Cu-TPBTM	L	L	L
IFMC-1	L	L	L

$$\mathrm{MSE} = \frac{1}{n}\sum_{i=1}^{n}(y_n - \hat{y}_n)^2,$$
$$\mathrm{AIC} = -\log(\mathrm{MSE}) - 2n - 2n\log(2\pi) + 2k,$$
$$\mathrm{BIC} = -\log(\mathrm{MSE}) - 2n - 2n\log(2\pi) + k\log(n),$$

Figure S3. Cross validation of the linear regression models. (a) (e) Linear regression without testing data for the models MNA/-/- and MNA/-/U, respectively. The same results as Figs. 5 (a) (b). (b) (c) (d) Linear regression results with 20 training data (filled circles) and predicted values for 14 testing data (open circles) based on the model MNA/-/-. (f) (g) (h) The results based on the model MNA/-/U. (i) Divided data sets G1, G2 and G3 for each of (b, f), (c, g) and (d, h). "L" and "P" represent training and testing data, respectively. The tables show adj R2, AIC and BIC scores for all the training and testing results. The definitions of AIC and BIC are shown above. Here, n and k represent the number of datas and the number of explanatory variables, respectively.

Materials	experimental values (%(w/v))	(a)	(b)	(c)	(d)	(e)	(f)	(g)	(h)
MOF-5	0.054	0.058	**0.063**	0.065	**0.066**	0.070	**0.069**	0.067	**0.065**
MIL-101	0.067	0.100	**0.111**	0.113	0.098	0.105	**0.111**	0.108	0.095
Mn-MOF-74	0.262	0.305	**0.327**	**0.309**	**0.311**	0.294	**0.317**	**0.302**	**0.304**
Mg-MOF-74	0.344	0.320	0.343	0.323	0.327	0.311	0.335	0.320	0.323
Fe-MOF-74	0.350	0.314	0.336	0.318	0.321	0.300	0.324	0.308	0.312
Zn-MOF-74	0.307	0.322	0.345	0.325	0.329	0.315	0.338	0.323	0.326
Ni-MOF-74	0.307	0.327	**0.350**	0.330	0.334	0.317	**0.341**	0.325	0.329
Co-MOF-74	0.286	0.319	**0.342**	0.323	0.326	0.314	**0.336**	0.323	0.325
MAF-23	0.171	0.187	0.171	**0.174**	0.216	0.155	0.150	**0.143**	0.184
bio-MOF-11	0.217	0.257	0.199	0.248	**0.185**	0.231	0.188	0.224	**0.130**
TMOF-1	0.086	0.161	0.163	0.142	**0.209**	0.143	0.148	0.129	**0.182**
UMCM-1	0.018	0.042	0.048	**0.053**	0.044	0.018	0.027	**0.017**	0.015
MOF-177	0.018	0.042	0.048	**0.053**	**0.044**	0.007	0.019	**0.006**	**0.006**
SNU-150	0.055	0.105	**0.109**	**0.100**	**0.132**	0.093	**0.097**	**0.085**	**0.110**
Zn-SIFSIX-3	0.173	0.166	**0.168**	**0.146**	**0.216**	0.166	**0.166**	**0.151**	**0.203**
Cu-SIFSIX-1	0.198	0.101	0.105	**0.097**	**0.126**	0.141	0.132	**0.134**	**0.146**
Cu-TATB	0.069	0.094	**0.096**	**0.106**	**0.091**	0.061	**0.070**	**0.062**	**0.059**
Cu-SIFSIX-2	0.078	0.079	**0.084**	0.081	**0.095**	0.049	**0.058**	0.044	**0.060**
Cu-TPBTM	0.149	0.135	0.142	0.142	0.138	0.126	0.133	0.127	0.125
ZJNU-54	0.177	0.232	0.207	0.239	0.177	0.255	0.228	0.259	0.177
rht-MOF-7	0.197	0.183	0.186	**0.189**	0.185	0.202	0.199	**0.203**	0.197
Nbo-Pd-1	0.262	0.172	0.184	0.176	0.183	0.201	0.203	0.202	0.201
UiO-66	0.123	0.123	0.126	0.114	0.156	0.159	0.150	0.149	0.172
Zn-BTZ	0.247	0.169	**0.147**	0.166	0.178	0.192	**0.167**	0.184	0.193
SNU-50	0.102	0.130	0.140	**0.135**	0.139	0.159	0.158	**0.159**	0.155
JLU-Lin21	0.167	0.168	0.173	0.175	**0.170**	0.162	0.168	0.164	**0.162**
PCN-124	0.190	0.145	**0.148**	**0.144**	0.161	0.154	**0.153**	**0.150**	0.160
Zn2-tdc-2	0.404	0.373	**0.245**	0.375	**0.154**	0.378	**0.267**	0.380	**0.092**
nbo-Cu	0.183	0.171	**0.184**	0.176	**0.180**	0.183	**0.190**	0.184	**0.184**
HKUST-1	0.216	0.211	0.226	**0.216**	0.218	0.230	0.238	**0.233**	0.230
Cu-Me	0.239	0.183	0.189	0.181	0.202	0.193	0.195	0.190	0.203
PCN-88	0.138	0.131	0.141	**0.138**	**0.138**	0.128	0.136	**0.128**	**0.129**
Cu-TDPAT	0.200	0.182	**0.185**	0.188	0.184	0.199	**0.197**	0.200	0.194
IFMC-1	0.149	0.194	0.153	0.196	0.183	0.191	0.159	0.185	0.184

Table S3. Predict values of crosss validation of the linear regression models (Fig. S3). Bold faced Values are "testing data" (open circles in Fig. S3).
